# Checklist of the fungus gnats of Finland: Bolitophilidae, Diadocidiidae, Ditomyiidae, Keroplatidae and Mycetophilidae (Diptera)

**DOI:** 10.3897/zookeys.441.7646

**Published:** 2014-09-19

**Authors:** Jevgeni Jakovlev

**Affiliations:** 1 Finnish Environment Institute, Helsinki, Finland

**Keywords:** Species list, Finland, Diptera, biodiversity, faunistics

## Abstract

A checklist of the ’fungus gnat’ group of families in Sciaroidea: Bolitophilidae, Diadocidiidae, Ditomyiidae, Keroplatidae and Mycetophilidae (Diptera) recorded from Finland. At present, the known Finnish fauna comprises 767 recognized species in 78 genera; 21 species of the family Bolitophilidae, 5 of the family Diadocidiidae, 2 of the family Ditomyiidae, 47 of the family Keroplatidae and 692 of the family Mycetophilidae. The unassigned genus *Sciarosoma* Chandler, 2002 is not included in this paper because it is listed with Sciaridae.

The most relevant synonyms, on which species were ever listed from Finland in Hackman (1980) and subsequent authors are indicated in the checklist. Eighteen species names of wrong records were removed.

## Introduction

Fungus gnats are a rich assemblage of nematocerous flies (Diptera, Nematocera) placed in the infraorder Bibionomorpha and superfamily Sciaroidea, currently divided into eight families (Blagoderov and Grimaldi 2004). Five of these families (Bolitophilidae, Diadocidiidae, Ditomyiidae, Keroplatidae and Mycetophilidae) are called informally ‘fungus gnats’ by most European authors. Species in these families are morphologically closely related and the group is also ecologically uniform.

Adults of fungus gnats are generally quite easily recognized by their habitus. They are slender to moderately robust flies between 2 and 10 mm (a few up to 15 mm) in body length, with long antenna, a high and often strongly arched thorax made distinctly higher by the long, stout and tight-sitting coxae of their legs. Their wings have reduced and variable venation that is quite distinctive for the different families and much used also to classify genera. Their body and legs are often covered with strong bristles. As larvae the majority of fungus gnat species are mycetophagous, associated with fungi, either fruiting bodies or mycelia in dead wood and soil litter.

Here I cover all the Finnish fungus gnat fauna of the families Bolitophilidae, Diadocidiidae, Ditomyiidae, Keroplatidae, and Mycetophilidae. In Europe, the superfamily Sciaroidea further includes two large families, the Sciaridae – black-winged fungus gnats and the Cecidomyiidae – gall midges (Søli et al. 2000) although some authors, e.g. Hippa and Vilkamaa (2006) consider the Cecidomyiidae as the sister group of all other Sciaroidea. They differ markedly in habitus and larval biology and are presented elsewhere. Finally, the unassigned genus *Sciarosoma* Chandler, 2002 is not included in this checklist since it is listed with the family Sciaridae according Hippa and Vilkamaa (2006), but see also opinion by Jaschhof et al. (2006).

Systematics and nomenclature of the enumeration follow the online checklist of Nordic fungus gnats (Kjærandsen 2012). Only the most relevant synonyms, on which species were ever listed from Finland, are included in the checklist.

Adults of the North European species can largely be identified using Hutson et al. (1980) and Zaitzev (1994) − Bolitophilidae, Diadocidiidae, Ditomyiidae, Keroplatidae, and Mycetophilidae in part (Mycomyiinae, Sciophilinae, Gnoristinae and Leiinae), and Zaitzev (2003) – Mycetophilidae in part (Mycetophilinae and Manotinae). However, since many new taxa are added, and corrections, synonyms and revisions continually published, one needs a complete set of the fragmented taxonomic literature to cover all the species. A good source for obtaining most important literature on the Sciaroidea is the ViBrant/Scratchpad module Fungus Gnats Online (2014). Species identification of fungus gnats based on examination of larvae is currently impossible because the larvae seemingly lack unique morphological characteristics and search for species-specific molecular markers are yet to be implemented.

Fungus gnats are distributed all over the world with about 5,300 known species (Table 1), more than 1,450 of them are known from the Palaearctic region (Søli et al. 2000). According to the current version of the Fauna Europaea Web-database (Chandler 2004), 1,098 species of fungus gnats had then been recorded from Europe. Taking into account literature published subsequently these figures could be increased in ca 100 additional species, i.e. some 1,200 species are known in Europe at present. As opposed to many other insect groups, fungus gnats, at least Mycetophilidae, seem to display an increasing diversity towards the north in Europe, and in the European perspective, they are especially diverse in the boreal zone. The current version of the checklist of North European (Sweden, Norway, Finland, Russian Karelia, Russian Lapland, Denmark, The Faroes, and Iceland) fungus gnats (Kjærandsen 2012) includes 896 species.

In Finland, 269 species of the fungus gnats (of which ca. 40 currently recognized species were described by Carl Lundström) were first listed in the Enumeration Insectorum Fenniae (Frey et al. 1941). Hackman (1980) presented the second checklist of Finnish Diptera that incorporates 486 species of fungus gnats including five with a question mark. This list of species, with minor additions and corrections concerning Mycetophilidae after systematical revisions of the genera *Trichonta* (Gagné 1981) and *Mycomya* (Väisänen 1984) in the Holarctic region was included into Catalogue of the Palaearctic Diptera (Hackman et al. 1988). During the recent two decades, large-scale inventories (e.g. Polevoi and Jakovlev 2004, Polevoi et al. 2006, Jakovlev et al. 2006, 2014, Jakovlev and Penttinen 2007) have increased the total number of Finnish fungus gnats with ca. 300 species. The 2010 Finnish Red List assessment of fungus gnats was based on a regional species pool consisting on 734 species (Penttinen et al. 2010). At present, the Finnish fungus gnat fauna incorporates 767 recognized species (except *Sciarosoma*) and ca. 20 species awaiting descriptions. Altogether, 18 species names of wrong records from Finland were removed from the checklist. These species and 3 doubtful species names are listed in the end.

**Table 1.** Number of species by family.

Family	Number of species (except *Sciarosoma*) in				Level of knowledge
	World (Pape et al. 2011)	Europe (Chandler 2004)	Nordic Region (Kjærandsen 2012)	Finland	
Bolitophilidae	59	36	31	21	average
Diadocidiidae	34	5	5	5	average
Ditomyiidae	94	4	3	2	average
Keroplatidae	953	110	62	47	average
Mycetophilidae	4150	942	794	692	average

## Checklist

Nematocera Dumeril, 1805

infraorder Bibionomorpha Hennig, 1954

superfamily Sciaroidea Schiner, 1864

**BOLITOPHILIDAE** Winnertz, 1864

***BOLITOPHILA*** Meigen, 1818

**sg. *Bolitophila*** Meigen, 1818

*Bolitophila austriaca* (Mayer, 1950)

*Bolitophila basicornis* (Mayer, 1951)

*Bolitophila caspersi* Plassmann, 1956

*Bolitophila cinerea* Meigen, 1818

*Bolitophila saundersii* (Curtis, 1836)

*Bolitophila tenella* Winnertz, 1864

**sg. *Cliopisa*** Enderlein, 1936

*Bolitophila aperta* Lundström, 1915

*Bolitophila bimaculata* Zetterstedt, 1838

*Bolitophila dubia* Siebke, 1863

= *disjuncta* Loew, 1869

*Bolitophila fumida* Edwards, 1941

*Bolitophila glabrata* Loew, 1869

*Bolitophila hybrida* (Meigen, 1804)

*Bolitophila ingrica* Stackelberg, 1969

*Bolitophila limitis* Polevoi, 1996

*Bolitophila maculipennis* Walker, 1836

= *coronata* Mayer, 1951

*Bolitophila modesta* Lackschewitz, 1937

= *tarsata* Mayer, 1951

*Bolitophila nigrolineata* Landrock, 1912

*Bolitophila obscurior* Stackelberg, 1969

*Bolitophila occlusa* Edwards, 1913

*Bolitophila pseudohybrida* Landrock, 1912

*Bolitophila rossica* Landrock, 1912

**DIADOCIDIIDAE** Winnertz, 1864

***DIADOCIDIA*** Ruthe, 1831

**sg. *Adidocidia*** Laštovka & Matile 1972

*Diadocidia trispinosa* Polevoi, 1996

*Diadocidia valida* Mik, 1874

**sg. *Diadocidia*** Ruthe, 1831

*Diadocidia ferruginosa* (Meigen, 1830)

*Diadocidia fissa* Zaitzev, 1994

*Diadocidia spinosula* Tollet, 1948

**DITOMYIIDAE** Keilin, 1919

***SYMMERUS*** Walker, 1848

*Symmerus annulatus* (Meigen, 1830)

*Symmerus nobilis* Lackschewitz, 1937

**KEROPLATIDAE** Rondani, 1856

KEROPLATINAE Rondani, 1856

tribe Keroplatini Rondani, 1856

***CEROTELION*** Rondani, 1856

*Cerotelion striatum* (Gmelin, 1790)

= *lineatum* (Fabricius, 1775) preocc.

***KEROPLATUS*** Bosc, 1792

*Keroplatus testaceus* Dalman, 1818

*Keroplatus tipuloides* Bosc, 1792

= *sesioides* Wahlberg, 1839

* *Keroplatus tuvensis* Zaitzev, 1991 (see Notes)

***ROCETELION*** Matile, 1988

*Rocetelion humerale* (Zetterstedt, 1850)

tribe Orfeliini Matile, 1990

***ISONEUROMYIA*** Brunetti, 1912

*Isoneuromyia semirufa* (Meigen, 1818)

***MACRORRHYNCHA*** Winnertz, 1846

*Macrorrhyncha flava* Winnertz, 1846

*Macrorrhyncha rostrata* (Zetterstedt, 1851)

***MONOCENTROTA*** Edwards, 1925

*Monocentrota lundstroemi* Edwards, 1925

***NEOPLATYURA*** Malloch, 1928

*Neoplatyura flava* (Macquart, 1826)

*Neoplatyura modesta* (Winnertz, 1864)

***ORFELIA*** Costa, 1857

*Orfelia discoloria* (Meigen, 1818)

*Orfelia falcata* Zaitzev, 1994

*Orfelia fasciata* (Meigen, 1804)

*Orfelia krivosheinae* Zaitzev, 1994

*Orfelia lugubris* (Zetterstedt, 1851)

= *tristis* (Lundström, 1911)

*Orfelia nemoralis* (Meigen, 1818)

*Orfelia nigricornis* (Fabricius, 1805)

*Orfelia ochracea* (Meigen, 1818)

= *unicolor* (Staeger, 1840)

*Orfelia pallida* (Staeger, 1840)

***PYRATULA*** Edwards, 1929

*Pyratula perpusilla* (Edwards, 1913)

*Pyratula subcanariae* Chandler, 2001

*Pyratula zonata* (Zetterstedt, 1855)

***URYTALPA*** Edwards, 1929

*Urytalpa atriceps* (Edwards, 1913)

*Urytalpa dorsalis* (Staeger, 1840)

= *ochracea* (Meigen, 1818)

*Urytalpa galdes* Hedmark & Kjærandsen, 2009

*Urytalpa macrocera* (Edwards, 1913)

*Urytalpa trivittata* (Lundström, 1914)

MACROCERINAE Rondani, 1856

tribe Macrocerini Rondani, 1856

***MACROCERA*** Meigen, 1803

*Macrocera angulata* Meigen, 1818

*Macrocera centralis* Meigen, 1818

*Macrocera crassicornis* Winnertz, 1864

*Macrocera estonica* Landrock, 1924

*Macrocera fasciata* Meigen, 1804

*Macrocera fascipennis* Staeger, 1840

*Macrocera grandis* Lundström, 1912

= *magna* Landrock, 1917

*Macrocera lutea* Meigen, 1804

*Macrocera maculata* Meigen, 1818

*Macrocera nigricoxa* Winnertz, 1864

*Macrocera parva* Lundström, 1914

*Macrocera phalerata* Meigen, 1818

*Macrocera pilosa* Landrock, 1917

*Macrocera pumilio* Loew, 1869

*Macrocera pusilla* Meigen, 1830

*Macrocera stigma* Curtis, 1837

*Macrocera stigmoides* Edwards, 1925

*Macrocera vittata* Meigen, 1830

*Macrocera zetterstedti* Lundström, 1914

= *nana* Zetterstedt, 1860

**MYCETOPHILIDAE** Newman, 1834

MYCOMYINAE Edwards, 1925

***MYCOMYA*** Rondani, 1856

**sg. *Calomycomya*** Väisänen, 1984

*Mycomya avala* Väisänen, 1984

*Mycomya pulchella* (Dziedzicki, 1885)

**sg. *Coheromyia*** Väisänen, 1984

*Mycomya branderi* Väisänen, 1984

= *tantilla* auct. nec (Loew, 1869) misid.

**sg. *Cymomya*** Väisänen, 1984

*Mycomya circumdata* (Staeger, 1840)

**sg. *Lycomya*** Väisänen, 1984

*Mycomya pectinifera* Edwards, 1924

**sg. *Mycomya*** Rondani, 1856

*Mycomya annulata* (Meigen, 1818)

= *incisurata* (Zetterstedt, 1838)

*Mycomya bialorussica* Landrock, 1925

*Mycomya bicolor* (Dziedzicki, 1885)

*Mycomya bisulca* Lackschewitz, 1937

*Mycomya britteni* Kidd, 1955

*Mycomya brunnea* (Dziedzicki, 1885)

*Mycomya cinerascens* (Macquart, 1826)

= *hyalinata* (Meigen, 1830)

*Mycomya collini* Edwards, 1941

*Mycomya danielae* Matile, 1972

*Mycomya denmax* Väisänen, 1979

*Mycomya digitifera* Edwards, 1925

*Mycomya disa* Väisänen, 1984

*Mycomya dziedzickii* Väisänen, 1984

= *fulva* (Dziedzicki, 1885) preocc.

*Mycomya egregia* (Dziedzicki, 1885)

*Mycomya festivalis* Väisänen, 1984

*Mycomya flavicollis* (Zetterstedt, 1852)

*Mycomya forestaria* Plassmann, 1978

*Mycomya fuscata* (Winnertz, 1864)

*Mycomya griseovittata* (Zetterstedt, 1852)

= *fasciata* (Zetterstedt, 1838) preocc.

= *clavigera* (Lundström, 1912)

*Mycomya hackmani* Väisänen, 1984

*Mycomya heydeni* Plassmann, 1970

*Mycomya hians* (Lundström, 1912)

*Mycomya hiisi* Väisänen, 1979

*Mycomya humida* Garrett, 1924

= *curvata* Fisher, 1937

*Mycomya indistincta* Polevoi, 1995

*Mycomya insignis* (Winnertz, 1864)

= *wrzesniowskii* (Dziedzicki, 1885)

*Mycomya islandica* Väisänen, 1984

*Mycomya karelica* Väisänen, 1979

*Mycomya kaurii* Väisänen, 1979

*Mycomya kuusamoensis* Väisänen, 1979

*Mycomya lambi* Edwards, 1941

*Mycomya leporina* Väisänen, 1984

*Mycomya levis* (Dziedzicki, 1885)

*Mycomya livida* (Dziedzicki, 1885)

*Mycomya maculata* (Meigen, 1804)

*Mycomya marginata* (Meigen, 1818)

= *limbata* (Winnertz, 1864)

*Mycomya mituda* Väisänen, 1980

*Mycomya neohyalinata* Väisänen, 1984

*Mycomya nigricornis* (Zetterstedt, 1852)

= *melanoceras* Edwards, 1924

*Mycomya nitida* (Zetterstedt, 1852)

= *exigua* (Winnertz, 1864)

*Mycomya norna* Väisänen, 1984

*Mycomya occultans* (Winnertz, 1864)

*Mycomya ornata* (Meigen, 1818)

*Mycomya parva* (Dziedzicki, 1885)

*Mycomya prominens* (Lundström, 1913)

*Mycomya pseudoapicalis* Landrock, 1925

*Mycomya pseudocurvata* Väisänen, 1979

*Mycomya punctata* (Meigen, 1804)

*Mycomya ruficollis* (Zetterstedt, 1852)

*Mycomya safena* Väisänen, 1984

*Mycomya shermani* Garrett, 1924

= *kingi* Edwards, 1941

*Mycomya shewelli* Väisänen, 1984

*Mycomya siebecki* (Landrock, 1912)

*Mycomya sieberti* (Landrock, 1930)

*Mycomya sigma* Johannsen, 1910

= *duplicata* Edwards, 1925

*Mycomya simulans* Väisänen, 1984

*Mycomya spinicoxa* Väisänen, 1979

*Mycomya storai* Väisänen, 1979

*Mycomya subarctica* Väisänen, 1979

*Mycomya tenuis* (Walker, 1856)

*Mycomya thula* Väisänen, 1984

*Mycomya trivittata* (Zetterstedt, 1838)

*Mycomya tumida* (Winnertz, 1864)

*Mycomya vittiventris* (Zetterstedt, 1852)

= *elegans* (Lundström, 1912)

*Mycomya wankowiczii* (Dziedzicki, 1885)

*Mycomya winnertzi* (Dziedzicki, 1885)

**sg. *Mycomyopsis*** Väisänen, 1984

*Mycomya affinis* (Staeger, 1840)

= *flava* (Winnertz, 1864)

*Mycomya confusa* Väisänen, 1977

*Mycomya fennica* Väisänen, 1979

*Mycomya maura* (Walker, 1856)

*Mycomya neolittoralis* Väisänen, 1984

*Mycomya paradentata* Väisänen, 1984

= *dentata* auct. nec Fisher, 1937 misid.

*Mycomya penicillata* (Dziedzicki, 1885)

*Mycomya permixta* Väisänen, 1984

*Mycomya trilineata* (Zetterstedt, 1838)

= *fusca* (Meigen, 1818)

**sg. *Neomycomya*** Väisänen, 1984

*Mycomya fimbriata* (Meigen, 1818)

***NEOEMPHERIA*** Osten Sacken, 1878

*Neoempheria bimaculata* (von Roser, 1840)

*Neoempheria pictipennis* (Haliday, 1833)

*Neoempheria striata* (Meigen, 1818)

*Neoempheria tuomikoskii* Väisänen, 1982

*Neoempheria winnertzi* Edwards, 1913

SCIOPHILINAE Rondani, 1840

***ACNEMIA*** Winnertz, 1864

*Acnemia amoena* Winnertz, 1864

*Acnemia angusta* Zaitzev, 1982

*Acnemia falcata* Zaitzev, 1982

*Acnemia longipes* Winnertz, 1864

*Acnemia nitidicollis* (Meigen, 1818)

*Acnemia trifida* Zaitzev, 1982

***ALLOCOTOCERA*** Mik, 1886

*Allocotocera pulchella* (Curtis, 1837)

***ANACLILEIA*** Meunier, 1904

*Anaclileia dispar* (Winnertz, 1864)

*Anaclileia dziedzickii* (Landrock, 1911)

***AZANA*** Walker, 1856

**sg. *Azana*** Walker, 1856

*Azana anomala* (Staeger, 1840)

***COELOPHTHINIA*** Edwards, 1941

*Coelophthinia thoracica* (Winnertz, 1864)

***EUDICRANA*** Loew, 1869

*Eudicrana nigriceps* (Lundström, 1909)

***LEPTOMORPHUS*** Curtis, 1831

**sg. *Leptomorphus*** Curtis, 1831

*Leptomorphus forcipatus* Landrock, 1918

*Leptomorphus subforcipatus* Zaitzev & Ševčík, 2002

= *quadrimaculatus* auct. nec Matsumura, 1910 misid. (see Notes)

*Leptomorphus walkeri* Curtis, 1831

***MEGALOPELMA*** Enderlein, 1911

*Megalopelma nigroclavatum* (Strobl, 1910)

***MONOCLONA*** Mik, 1886

*Monoclona braueri* (Strobl, 1895)

*Monoclona rufilatera* (Walker, 1837)

*Monoclona silvatica* Zaitzev, 1983

= *mikii* Kertesz,1898 misid. (see Notes)

***NEURATELIA*** Rondani, 1856

*Neuratelia nemoralis* (Meigen, 1818)

*Neuratelia sintenisi* Lackschewitz, 1937

***PARATINIA*** Mik, 1874

*Paratinia sciarina* Mik, 1874

***PHTHINIA*** Winnertz, 1864

*Phthinia congenita* Plassmann, 1984

*Phthinia humilis* Winnertz, 1864

*Phthinia mira* (Ostroverkhova, 1977)

= *plassmanni* Caspers, 1984

*Phthinia setosa* Zaitzev, 1994

*Phthinia winnertzi* Mik, 1869

***POLYLEPTA*** Winnertz, 1864

*Polylepta borealis* Lundström, 1912

*Polylepta guttiventris* (Zetterstedt, 1852)

***SCIOPHILA*** Meigen, 1818

*Sciophila adamsi* Edwards, 1925

*Sciophila antiqua* Chandler, 1987

= *hebes* auct. nec Johannsen, 1910 misid. (see Notes)

*Sciophila bicuspidata* Zaitzev, 1982

*Sciophila buxtoni* Freeman, 1956

*Sciophila caesarea* Chandler, 2001

*Sciophila dziedzickii* Edwards, 1925

*Sciophila fenestella* Curtis, 1837

*Sciophila fuliginosa* Holmgren, 1883

*Sciophila geniculata* Zetterstedt, 1838

*Sciophila hirta* Meigen, 1818

*Sciophila karelica* Zaitzev, 1982

*Sciophila krysheni* Polevoi, 2001

*Sciophila limbatella* Zetterstedt, 1852

*Sciophila lutea* Macquart, 1826

*Sciophila minuta* Zaitzev, 1982

*Sciophila modesta* Zaitzev, 1982

*Sciophila nigronitida* Landrock, 1925

*Sciophila nonnisilva* Hutson, 1979

*Sciophila persubtilis* Polevoi, 2001

*Sciophila plurisetosa* Edwards, 1921

*Sciophila pomacea* Chandler, 2006

= *ochracea* Stephens in Walker, 1856 preocc.

*Sciophila pseudoflexuosa* Kurina, 1991

*Sciophila rufa* Meigen, 1830

*Sciophila salassea* Matile, 1983

*Sciophila setosa* Garrett, 1925

*Sciophila thoracica* Staeger, 1840

= *quadriterga* (Hutson, 1979)

*Sciophila varia* (Winnertz, 1864)

*Sciophila yakutica* Blagoderov, 1992

GNORISTINAE Edwards, 1925

***ACOMOPTERA*** Vockeroth, 1980

*Acomoptera difficilis* (Dziedzicki 1885)

*Acomoptera spinistylus* (Søli, 1993)

***APOLEPHTHISA*** Grzegorzek, 1885

*Apolephthisa subincana* (Curtis, 1837)

***BOLETINA*** Staeger, 1840

*Boletina atridentata* Polevoi & Hedmark, 2004

*Boletina basalis* (Meigen, 1818)

*Boletina bidenticulata* Sasakawa & Kimura, 1974

*Boletina borealis* Zetterstedt, 1852

*Boletina brevicornis* Zetterstedt, 1852

*Boletina cincticornis* (Walker, 1848)

*Boletina cordata* Polevoi & Hedmark, 2004

*Boletina cornuta* Zaitzev, 1994

*Boletina digitata* Lundström, 1914

*Boletina dispecta* Dziedzicki, 1885

*Boletina dispectoides* Jakovlev & Penttinen, 2007

*Boletina dissipata* Plassmann, 1986

*Boletina dubia* (Meigen, 1804)

*Boletina edwardsi* Chandler, 1992

*Boletina falcata* Polevoi & Hedmark, 2004

*Boletina fennoscandica* Polevoi & Hedmark, 2004

*Boletina gripha* Dziedzicki, 1885

*Boletina griphoides* Edwards, 1925

*Boletina groenlandica* Staeger, 1845

*Boletina gusakovae* Zaitzev, 1994

*Boletina hedstroemi* Polevoi & Hedmark, 2004

*Boletina intermedia* Lundström, 1915

*Boletina jamalensis* Zaitzev, 1994

= *struthioides* Polevoi & Hedmark, 2004

*Boletina kivachiana* Polevoi & Hedmark, 2004

*Boletina kurilensis* Zaitzev, 1994

*Boletina landrocki* Edwards, 1924

*Boletina lapponica* Polevoi & Hedmark, 2004

*Boletina lundbecki* Lundström, 1912

*Boletina lundstroemi* Landrock, 1912

*Boletina maculata* Holmgren, 1870

= *apicalis* (Walker, 1848) preocc.

*Boletina minuta* Polevoi in Zaitzev & Polevoi, 1995

*Boletina moravica* Landrock, 1912

*Boletina nasuta* (Haliday, 1839)

*Boletina nigricans* Dziedzicki, 1885

*Boletina nigricoxa* Staeger, 1840

*Boletina nigrofusca* Dziedzicki, 1885

*Boletina nitida* Grzegorzek, 1885

*Boletina nitiduloides* Zaitzev, 1994

*Boletina onegensis* Polevoi in Zaitzev & Polevoi, 1995

*Boletina pallidula* Edwards, 1925

*Boletina palmata* Polevoi, 2013

*Boletina pectinunguis* Edwards, 1932

*Boletina pinusia* Maximova, 2001

*Boletina plana* (Walker, 1856)

*Boletina polaris* Lundström, 1915

*Boletina populina* Polevoi in Zaitzev & Polevoi, 1995

*Boletina pseudonitida* Zaitzev, 1994

*Boletina rejecta* Edwards, 1941

*Boletina sciarina* Staeger, 1840

*Boletina silvatica* Dziedzicki, 1885

*Boletina subtriangularis* Polevoi & Hedmark, 2004

*Boletina takagii* Sasakawa & Kimura, 1974

*Boletina tiroliensis* Plassmann, 1980

*Boletina triangularis* Polevoi in Zaitzev & Polevoi, 1995

*Boletina trispinosa* Edwards, 1913

*Boletina trivittata* (Meigen, 1818)

*Boletina verticillata* Stackelberg, 1943

*Boletina villosa* Landrock, 1912

***COELOSIA*** Winnertz, 1864

*Coelosia bicornis* Stackelberg, 1946

*Coelosia flava* (Staeger, 1840)

*Coelosia fusca* Bezzi, 1892

= *silvatica* Landrock, 1918

*Coelosia gracilis* Johannsen, 1912

*Coelosia limpida* Plassmann, 1986

*Coelosia tenella* (Zetterstedt, 1852)

*Coelosia truncata* Lundström, 1909

***DZIEDZICKIA*** Johannsen, 1909

*Dziedzickia marginata* (Dziedzicki, 1885)

***ECTREPESTHONEURA*** Enderlein, 1911

*Ectrepesthoneura colyeri* Chandler, 1980

*Ectrepesthoneura hirta* (Winnertz, 1846)

*Ectrepesthoneura nigra* Zaitzev, 1984

*Ectrepesthoneura ovata* Ostroverkhova, 1977

= *bucera* Plassmann, 1980

*Ectrepesthoneura pubescens* (Zetterstedt, 1860)

*Ectrepesthoneura referta* Plassmann, 1976

*Ectrepesthoneura tori* Zaitzev & Økland, 1994

***GNORISTE*** Meigen, 1818

*Gnoriste apicalis* Meigen, 1818

*Gnoriste bilineata* Zetterstedt, 1852

***GRZEGORZEKIA*** Edwards, 1941

*Grzegorzekia collaris* (Meigen, 1818)

***HADRONEURA*** Lundström, 1906

*Hadroneura palmeni* Lundström, 1906

***IMPLETA*** Plassmann, 1978

*Impleta consorta* Plassmann, 1978

***KATATOPYGIA*** Martinsson & Kjærandsen, 2012

*Katatopygia erythropyga* (Holmgren, 1883)

*Katatopygia sahlbergi* (Lundström, 1906)

***PALAEODOCOSIA*** Meunier, 1904

*Palaeodocosia vittata* (Coquillett, 1901)

= *janickii* (Dziedzicki, 1923)

= *alpicola* auct. nec (Strobl, 1894) misid. (see Notes)

***SAIGUSAIA*** Vockeroth, 1980

*Saigusaia flaviventris* (Strobl, 1894)

***SPEOLEPTA*** Edwards, 1925

*Speolepta leptogaster* (Winnertz, 1864)

***SYNAPHA*** Meigen, 1818

*Synapha fasciata* Meigen, 1818

*Synapha vitripennis* (Meigen, 1818)

***SYNTEMNA*** Winnertz, 1864

*Syntemna daisetsuzana* Okada, 1938

*Syntemna elegantia* Plassmann, 1978

*Syntemna hungarica* (Lundström, 1912)

*Syntemna morosa* Winnertz, 1864

*Syntemna nitidula* Edwards, 1925

*Syntemna oulankaensis* Polevoi, 2003

*Syntemna penicilla* Hutson, 1979

*Syntemna relicta* (Lundström, 1912)

*Syntemna setigera* (Lundström, 1914)

= *haagvari* Økland, 1995

*Syntemna stylata* Hutson, 1979

*Syntemna stylatoides* Zaitzev, 1994

***TETRAGONEURA*** Winnertz, 1846

*Tetragoneura ambigua* Grzegorzek, 1885

*Tetragoneura obirata* Plassmann, 1990

*Tetragoneura pudogensis* Polevoi & Jakovlev, 2011

*Tetragoneura ruuhijarvii* Polevoi & Jakovlev, 2011

*Tetragoneura sylvatica* (Curtis, 1837)

LEIINAE Edwards, 1925

***CLASTOBASIS*** Skuse, 1890

*Clastobasis alternans* (Winnertz, 1864)

***DOCOSIA*** Winnertz, 1864

*Docosia expectata* Laštovka & Ševčík, 2006

*Docosia flavicoxa* Strobl, 1900

= *pallipes* Edwards, 1941

*Docosia gilvipes* (Haliday in Walker, 1856)

*Docosia landrocki* Laštovka & Ševčík, 2006

*Docosia muelleri* Plassmann, 1986

*Docosia tibialis* Laštovka & Ševčík, 2006

***GREENOMYIA*** Brunetti, 1912

*Greenomyia baikalica* Zaitzev, 1994

*Greenomyia borealis* (Winnertz, 1864)

*Greenomyia mongolica* Laštovka & Matile, 1974

***LEIA*** Meigen, 1818

*Leia bilineata* (Winnertz, 1864)

*Leia bimaculata* (Meigen, 1804)

*Leia crucigera* Zetterstedt, 1838

*Leia cylindrica* (Winnertz, 1864)

*Leia fascipennis* Meigen, 1818

*Leia longiseta* Barendrecht, 1938

*Leia picta* Meigen, 1818

*Leia subfasciata* (Meigen, 1818)

*Leia winthemii* Lehmann, 1822

***RONDANIELLA*** Johannsen, 1909

*Rondaniella dimidiata* (Meigen, 1804)

MANOTINAE Edwards, 1925

***MANOTA*** Williston, 1896

*Manota unifurcata* Lundström, 1913

MYCETOPHILINAE Newman, 1834

tribe Exechiini Edwards, 1925

***ALLODIA*** Winnertz, 1864

**sg. *Allodia*** Winnertz, 1864

*Allodia anglofennica* Edwards, 1921

*Allodia confusa* Zaitzev, 2003

= *simplex* Zaitzev, 1983 preocc.

*Allodia embla* Hackman, 1971

*Allodia lugens* (Wiedemann, 1817)

*Allodia lundstroemi* Edwards, 1921

*Allodia ornaticollis* (Meigen, 1818)

*Allodia pyxidiiformis* Zaitzev, 1983

*Allodia septentrionalis* Hackman, 1971

*Allodia truncata* Edwards, 1921

*Allodia tuomikoskii* Hackman, 1971

*Allodia zaitzevi* Kurina, 1998

**sg. *Brachycampta*** Winnertz, 1864

*Allodia adunca* Zaitzev, 1992

*Allodia alternans* (Zetterstedt, 1838)

*Allodia angulata* (Lundström, 1913)

*Allodia barbata* (Lundström, 1909)

*Allodia bohemica* Ševčík, 2004

*Allodia czernyi* (Landrock, 1912)

*Allodia foliifera* (Strobl, 1910)

*Allodia grata* (Meigen, 1830)

*Allodia huggerti* Kjærandsen, 2007

*Allodia neglecta* Edwards, 1925

*Allodia penicillata* (Lundström, 1912)

*Allodia pistillata* (Lundström, 1911)

*Allodia protenta* Laštovka & Matile, 1974

= *mendli* Plassmann, 1977

*Allodia silvatica* (Landrock, 1912)

*Allodia subpistillata* Ševčík, 1999

***ALLODIOPSIS*** Tuomikoski, 1966

*Allodiopsis domestica* (Meigen, 1830)

*Allodiopsis gracai* Ševčík & Papp 2003

*Allodiopsis korolevi* Zaitzev, 1982

*Allodiopsis pseudodomestica* (Lackschewitz, 1937)

*Allodiopsis rustica* (Edwards, 1941)

***ANATELLA*** Winnertz, 1864

*Anatella ankeli* Plassmann, 1977

*Anatella aquila* Zaitzev, 1989

*Anatella bremia* Chandler, 1994

*Anatella ciliata* Winnertz, 1864

*Anatella crispa* Zaitzev, 1994

*Anatella dampfi* Landrock, 1924

*Anatella dentata* Zaitzev, 1989

*Anatella emergens* Caspers, 1987

*Anatella flavicauda* Winnertz, 1864

*Anatella flavomaculata* Edwards, 1925

*Anatella gibba* Winnertz, 1864

*Anatella lenis* Dziedzicki, 1923

*Anatella maritima* Ostroverkhova, 1979

*Anatella minuta* (Staeger, 1840)

*Anatella novata* Dziedzicki, 1923

*Anatella setigera* Edwards, 1921

*Anatella simpatica* Dziedzicki, 1923

*Anatella turi* Dziedzicki, 1923

*Anatella unguigera* Edwards, 1921

***BRACHYPEZA*** Winnertz, 1864

**sg. *Brachypeza*** Winnertz, 1864

*Brachypeza armata* Winnertz, 1864

*Brachypeza bisignata* Winnertz, 1864

= *hilaris* Winnertz, 1864 preocc.

**sg. *Paracordyla*** Tuomikoski, 1966

*Brachypeza obscura* Winnertz, 1864

***BREVICORNU*** Marshall, 1896

*Brevicornu arcticoides* Caspers, 1985

*Brevicornu arcticum* (Lundström, 1913)

*Brevicornu auriculatum* (Edwards, 1925)

*Brevicornu beatum* (Johannsen, 1912)

*Brevicornu bellum* (Johannsen, 1912)

*Brevicornu bipartitum* Laštovka & Matile, 1974

*Brevicornu canescens* (Zetterstedt, 1852)

= *griseolum* auct. nec (Zetterstedt, 1852)

*Brevicornu cognatum* Ostroverkhova, 1979

*Brevicornu fasciculatum* (Lackschewitz, 1937)

*Brevicornu fennicum* (Landrock, 1927)

*Brevicornu fissicauda* (Lundström, 1911)

*Brevicornu foliatum* (Edwards, 1925)

*Brevicornu fuscipenne* (Staeger, 1840)

*Brevicornu glandis* Laštovka & Matile, 1974

*Brevicornu griseicolle* (Staeger, 1840)

*Brevicornu griseolum* (Zetterstedt, 1852)

= *boreale* (Lundström, 1914)

*Brevicornu improvisum* Zaitzev, 1992

*Brevicornu kingi* (Edwards, 1925)

*Brevicornu luteum* (Landrock, 1925)

*Brevicornu melanderi* Zaitzev, 1988

*Brevicornu nigrofuscum* (Lundström, 1909)

*Brevicornu occidentale* Zaitzev, 1988

*Brevicornu parafennicum* Zaitzev in Zaitzev & Polevoi, 1995

*Brevicornu proximum* (Staeger, 1840)

*Brevicornu rosmellitum* Chandler, 2001

*Brevicornu ruficorne* (Meigen, 1838)

*Brevicornu serenum* (Winnertz, 1864)

*Brevicornu sericoma* (Meigen, 1830)

*Brevicornu setulosum* Zaitzev, 1988

*Brevicornu spathulatum* (Lundström, 1911)

*Brevicornu verralli* (Edwards, 1925)

***CORDYLA*** Meigen, 1803

*Cordyla brevicornis* (Staeger, 1840)

*Cordyla crassicornis* Meigen, 1818

*Cordyla fasciata* Meigen, 1830

*Cordyla fissa* Edwards, 1925

*Cordyla flaviceps* (Staeger, 1840)

*Cordyla fusca* Meigen, 1804

*Cordyla insons* Laštovka & Matile, 1974

*Cordyla murina* Winnertz, 1864

*Cordyla nitens* Winnertz, 1864

*Cordyla nitidula* Edwards, 1925

= *bergensis* (Barendrecht, 1938)

*Cordyla parvipalpis* Edwards, 1925

*Cordyla pusilla* Edwards, 1925

= *sixi* (Barendrecht, 1938)

*Cordyla semiflava* (Staeger, 1840)

***EXECHIA*** Winnertz, 1864

*Exechia bicincta* (Staeger, 1840)

*Exechia borealis* Lundström, 1912

*Exechia cincta* Winnertz, 1864

*Exechia confinis* Winnertz, 1864

*Exechia contaminata* Winnertz, 1864

*Exechia cornuta* Lundström, 1914

*Exechia dentata* Lundström, 1916

*Exechia dizona* Edwards, 1924

*Exechia dorsalis* (Staeger, 1840)

*Exechia exigua* Lundström, 1909

*Exechia festiva* Winnertz, 1864

*Exechia frigida* (Boheman, 1865)

*Exechia fusca* (Meigen, 1804)

*Exechia lucidula* (Zetterstedt, 1838)

*Exechia lundstroemi* Landrock, 1923

*Exechia macula* Chandler, 2001

= *maculipennis* (Stannius, 1831) preocc.

*Exechia micans* Laštovka & Matile, 1974

*Exechia nigra* Edwards, 1925

*Exechia nigrofusca* Lundström, 1909

*Exechia nigroscutellata* Landrock, 1912

*Exechia papyracea* Stackelberg, 1948

*Exechia parva* Lundström, 1909

*Exechia parvula* (Zetterstedt, 1852)

= *nana* Staeger, 1840 preocc.

*Exechia pectinivalva* Stackelberg, 1948

*Exechia pseudocincta* Strobl, 1910

*Exechia pseudofestiva* Lackschewitz, 1937

*Exechia repanda* Johannsen, 1912

*Exechia repandoides* Caspers, 1984

*Exechia separata* Lundström, 1912

*Exechia seriata* (Meigen, 1830)

= *pallida* Stannius, 1831

*Exechia similis* Laštovka & Matile, 1974

*Exechia spinigera* Winnertz, 1864

*Exechia spinuligera* Lundström, 1912

*Exechia styriaca* Strobl, 1898

= *sororcula* Lackschewitz, 1937

*Exechia subfrigida* Laštovka & Matile, 1974

*Exechia unifasciata* Lackschewitz, 1937

*Exechia unimaculata* (Zetterstedt, 1860)

***EXECHIOPSIS*** Tuomikoski, 1966

**sg. *Exechiopsis*** Tuomikoski, 1966

*Exechiopsis aemula* Plassmann, 1984

*Exechiopsis clypeata* (Lundström, 1911)

*Exechiopsis distendens* (Lackschewitz, 1937)

*Exechiopsis dumitrescae* (Burghele-Balacesco, 1972)

*Exechiopsis fimbriata* (Lundström, 1909)

*Exechiopsis forcipata* Lackschewitz, 1937)

*Exechiopsis grassatura* (Plassmann, 1978)

*Exechiopsis hammi* (Edwards, 1925)

*Exechiopsis indecisa* (Walker, 1856)

*Exechiopsis ingrica* Stackelberg, 1948

*Exechiopsis intersecta* (Meigen, 1818)

*Exechiopsis januarii* (Lundström, 1913)

*Exechiopsis lackschewitziana* (Stackelberg, 1948)

*Exechiopsis landrocki* (Lundström, 1912)

*Exechiopsis ligulata* (Lundström, 1913)

*Exechiopsis pseudindecisa* Laštovka & Matile, 1974

*Exechiopsis pseudopulchella* (Lundström, 1912)

*Exechiopsis pulchella* (Winnertz, 1864)

*Exechiopsis sagittata* Laštovka & Matile, 1974

*Exechiopsis subulata* (Winnertz, 1864)

**sg. *Xenexechia*** Tuomikoski, 1966

*Exechiopsis crucigera* (Lundström, 1909)

*Exechiopsis davatchii* Matile, 1969

*Exechiopsis leptura* (Meigen, 1830)

*Exechiopsis membranacea* (Lundström, 1912)

*Exechiopsis perspicua* (Johannsen, 1912)

*Exechiopsis pollicata* (Edwards, 1925)

*Exechiopsis praedita* (Plassmann, 1976)

*Exechiopsis seducta* (Plassmann, 1976)

*Exechiopsis stylata* Laštovka & Matile, 1974

***MYROSIA*** Tuomikoski, 1966

*Myrosia maculosa* (Meigen, 1818)

***NOTOLOPHA*** Tuomikoski, 1966

*Notolopha brachycera* (Zetterstedt, 1852)

= *tuomikoskii* (Zaitzev & Maximova, 2000)

*Notolopha cristata* (Staeger, 1840)

*Notolopha sibirica* (Zaitzev & Maximova, 2000)

***PSEUDEXECHIA*** Tuomikoski, 1966

*Pseudexechia aurivernica* Chandler, 1978

*Pseudexechia canalicula* (Johannsen, 1912)

*Pseudexechia parallela* (Edwards, 1925)

*Pseudexechia pectinacea* (Ostroverkhova, 1979)

*Pseudexechia tristriata* Stackelberg, 1969

*Pseudexechia trivittata* (Staeger, 1840)

***PSEUDOBRACHYPEZA*** Tuomikoski, 1966

*Pseudobrachypeza helvetica* (Walker, 1856)

***PSEUDORYMOSIA*** Tuomikoski, 1966

*Pseudorymosia fovea* (Dziedzicki, 1910)

= *optiva* auct. nec (Dziedzicki, 1910) misid. (see Notes)

***RYMOSIA*** Winnertz, 1864

*Rymosia acta* Dziedzicki, 1910

*Rymosia affinis* Winnertz, 1864

= *gracilipes* Dziedzicki, 1910

*Rymosia batava* (Barendrecht, 1938)

*Rymosia bifida* Edwards, 1925

*Rymosia britteni* Edwards, 1925

*Rymosia connexa* Winnertz, 1864

*Rymosia fasciata* (Meigen, 1804)

*Rymosia fraudatrix* Dziedzicki, 1910

? = *spiniforceps* Matile, 1963

*Rymosia guttata* Lundström, 1912

*Rymosia pinnata* Ostroverkhova, 1979

*Rymosia placida* Winnertz, 1864

*Rymosia setiger* Dziedzicki, 1910

*Rymosia signatipes* (van der Wulp, 1859)

= *winnertzi* Barendrecht, 1938

***STIGMATOMERIA*** Tuomikoski, 1966

*Stigmatomeria crassicornis* (Stannius, 1831)

***SYNPLASTA*** Skuse, 1890

= *Gymnogonia* Tuomikoski, 1966

*Synplasta bayardi* Matile, 1971

*Synplasta dulcia* (Dziedzicki, 1910)

*Synplasta exclusa* (Dziedzicki, 1910)

= *sintenisi* (Lackschewitz, 1937)

*Synplasta gracilis* Winnertz, 1864

= *excogitata* auct. nec (Dziedzicki, 1910) misid. (see Notes)

*Synplasta ingeniosa* (Kidd 1969)

*Synplasta praeformida* (Dziedzicki, 1910)

*Synplasta pseudingeniosa* Zaitzev, 1993

*Synplasta rufilatera* (Edwards, 1941)

***TARNANIA*** Tuomikoski, 1966

*Tarnania fenestralis* (Meigen, 1838)

*Tarnania tarnanii* (Dziedzicki, 1910)

tribe Mycetophilini Newman, 1834

***DYNATOSOMA*** Winnertz, 1864

*Dynatosoma cochleare* Strobl, 1895

*Dynatosoma dihaeta* Polevoi, 1995

= *schachti* Plassmann, 1999

*Dynatosoma fuscicorne* (Meigen, 1818)

*Dynatosoma majus* Landrock, 1912

*Dynatosoma nigromaculatum* Lundström, 1913

= *abdominale* auct. nec Staeger, 1840

*Dynatosoma nobile* Loew, 1873

*Dynatosoma reciprocum* (Walker, 1848)

*Dynatosoma rufescens* (Zetterstedt, 1838)

= *bukowskii* Zaitzev, 1986

= *lutescens* (Zetterstedt, 1852)

*Dynatosoma silesiacum* Ševčík, 2001

*Dynatosoma thoracicum* (Zetterstedt, 1838)

= *norwegiense* Zaitzev & Økland, 1994

***EPICYPTA*** Winnertz, 1864

*Epicypta aterrima* (Zetterstedt, 1852)

*Epicypta fumigata* (Dziedzicki, 1923)

*Epicypta limnophila* Chandler, 1981

*Epicypta scatophora* (Perris, 1849)

***MYCETOPHILA*** Meigen, 1803

= *FUNGIVORA* Meigen, 1800 suppr.

*Mycetophila abbreviata* Landrock, 1914

*Mycetophila abiecta* (Laštovka, 1963)

*Mycetophila adumbrata* Mik, 1884

*Mycetophila alea* Laffoon, 1965

= *guttata* Dziedzicki, 1884

*Mycetophila attonsa* (Laffoon, 1957)

= *uncta* Plassmann, 1999

*Mycetophila autumnalis* Lundström, 1909

*Mycetophila bialorussica* Dziedzicki, 1884

* *Mycetophila biformis* Maximova, 2002 preocc. (see Notes)

*Mycetophila biusta* Meigen, 1818

*Mycetophila blanda* Winnertz, 1864

*Mycetophila bohemica* (Laštovka, 1963)

*Mycetophila boreocruciator* Ševčík, 2003

*Mycetophila brevitarsata* (Laštovka, 1963)

*Mycetophila caudata* Staeger, 1840

*Mycetophila cingulum* Meigen, 1830

*Mycetophila confluens* Dziedzicki, 1884

= *fulva* Winnertz, 1864

*Mycetophila confusa* Dziedzicki, 1884

= *affluctata* Edwards, 1941

*Mycetophila curviseta* Lundström, 1911

*Mycetophila deflexa* Chandler, 2001

*Mycetophila dentata* Lundström, 1915

*Mycetophila devioides* Bechev, 1988

*Mycetophila distigma* Meigen, 1830

= *w-fuscum* Dziedzicki, 1884

*Mycetophila dziedzickii* Chandler, 1977

= *obscura* Dziedzicki, 1884 nec Walker, 1848

*Mycetophila edwardsi* Lundström, 1913

*Mycetophila estonica* Kurina, 1992

*Mycetophila evanida* Laštovka, 1972

*Mycetophila exstincta* Loew, 1869

= *mikii* Dziedzicki, 1884

*Mycetophila finlandica* Edwards, 1913

*Mycetophila flava* Winnertz, 1864

*Mycetophila forcipata* Lundström, 1913

*Mycetophila formosa* Lundström, 1911

*Mycetophila freyii* Lundström, 1909

*Mycetophila fungorum* (De Geer, 1776)

*Mycetophila gibbula* Edwards, 1925

*Mycetophila hetschkoi* Landrock, 1918

*Mycetophila ichneumonea* Say, 1823

*Mycetophila idonea* Laštovka, 1972

*Mycetophila immaculata* (Dziedzicki, 1884)

*Mycetophila laeta* Walker, 1848

= *calva* Lundström, 1912

*Mycetophila lapponica* Lundström, 1906

*Mycetophila lobulata* Zaitzev, 1999

*Mycetophila lubomirskii* Dziedzicki, 1884

*Mycetophila luctuosa* Meigen, 1830

= *modesta* Winnertz, 1864

*Mycetophila magnicauda* Strobl, 1895

*Mycetophila marginata* Winnertz, 1864

*Mycetophila mitis* (Johannsen, 1912)

*Mycetophila mohilevensis* Dziedzicki, 1884

*Mycetophila monstera* Maximova, 2002

*Mycetophila morata* Zaitzev, 1999

*Mycetophila moravica* Landrock, 1925

*Mycetophila morosa* Winnertz, 1864

*Mycetophila nigrofusca* Dziedzicki, 1884

*Mycetophila occultans* Lundström, 1913

= *tarsata* Winnertz, 1864 preocc.

*Mycetophila ocellus* Walker, 1848

*Mycetophila ornata* Stephens, 1846

*Mycetophila ostentanea* Zaitzev, 1998

*Mycetophila pecinai* (Laštovka, 1963)

*Mycetophila perpallida* Chandler, 1993

*Mycetophila pictula* Meigen, 1830

= *bimaculata* Fabricius, 1805 nec Meigen, 1804

*Mycetophila pseudoforcipata* Zaitzev, 1998

*Mycetophila pumila* Winnertz, 1864

*Mycetophila pyrenaica* Matile, 1967

*Mycetophila quadra* Lundström, 1909

*Mycetophila ruficollis* Meigen, 1818

*Mycetophila schnabli* (Dziedzicki, 1884)

*Mycetophila sigmoides* Loew, 1869

*Mycetophila signata* Meigen, 1830

*Mycetophila signatoides* Dziedzicki, 1884

= *assimilis* Matile, 1967

*Mycetophila sinuosa* Plassmann & Schacht, 1999

*Mycetophila sordida* van der Wulp, 1874

*Mycetophila stolida* Walker, 1856

*Mycetophila stricklandi* (Laffoon, 1957)

*Mycetophila strigata* Staeger, 1840

*Mycetophila strigatoides* Landrock, 1927

*Mycetophila strobli* Laštovka, 1972

*Mycetophila stylata* (Dziedzicki, 1884)

*Mycetophila sublunata* Zaitzev, 1998

*Mycetophila subsigillata* Zaitzev, 1999

*Mycetophila sumavica* (Laštovka, 1963)

*Mycetophila triangularis* Lundström, 1912

*Mycetophila trinotata* Staeger, 1840

*Mycetophila uliginosa* Chandler, 1988

*Mycetophila unguiculata* Lundström, 1913

*Mycetophila uninotata* Zetterstedt, 1852

*Mycetophila unipunctata* Meigen, 1818

*Mycetophila xanthopyga* Winnertz, 1864

*Mycetophila zetterstedtii* Lundström, 1906

***PHRONIA*** Winnertz, 1864

*Phronia aviculata* Lundström, 1914

*Phronia avidoides* Jakovlev & Polevoi, 2009

*Phronia biarcuata* (Becker, 1908)

= *johannae* Steenberg, 1924

= *praecox* Edwards, 1925

*Phronia bicolor* Dziedzicki, 1889

= *fusciventris* van Duzee, 1928

*Phronia borealis* Hackman, 1970

*Phronia braueri* Dziedzicki, 1889

= *annulata* Winnertz, 1864

*Phronia caliginosa* Dziedzicki, 1889

= *trivittata* Dziedzicki, 1889

*Phronia cinerascens* Winnertz, 1864

*Phronia conformis* (Walker, 1856)

= *girschneri* Dziedzicki, 1889

*Phronia cordata* Lundström, 1914

*Phronia coritanica* Chandler, 1992

*Phronia cornuta* Lundström, 1914

*Phronia crassitarsus* Hackman, 1970

*Phronia digitata* Hackman, 1970

*Phronia disgrega* Dziedzicki, 1889

*Phronia distincta* Hackman, 1970

*Phronia dziedzickii* Lundström, 1906

*Phronia egregia* Dziedzicki, 1889

*Phronia electa* Dziedzicki, 1889

*Phronia elegans* Dziedzicki, 1889

*Phronia elegantula* Hackman, 1970

*Phronia exigua* (Zetterstedt, 1852)

*Phronia fennica* Jakovlev & Polevoi, 2009

*Phronia flavipes* Winnertz, 1864

*Phronia forcipata* Winnertz, 1864

*Phronia forcipula* Winnertz, 1864

*Phronia gagnei* Chandler, 1992

*Phronia gracilis* Hackman, 1970

*Phronia humeralis* Winnertz, 1864

*Phronia interstincta* Dziedzicki, 1889

*Phronia longelamellata* Strobl, 1898

= *minuta* Landrock, 1928

*Phronia lutescens* Hackman, 1970

*Phronia maculata* Dziedzicki, 1889

*Phronia mutabilis* Dziedzicki, 1889

*Phronia mutila* Lundström, 1911

*Phronia nigricornis* (Zetterstedt, 1852)

= *dubia* Dziedzicki, 1889

*Phronia nigripalpis* Lundström, 1909

*Phronia nitidiventris* (van der Wulp, 1859)

= *squalida* Winnertz, 1864

= *vitiosa* Winnertz, 1864

*Phronia notata* Dziedzicki, 1889

*Phronia obscura* Dziedzicki, 1889

*Phronia obtusa* Winnertz, 1864

*Phronia peculiaris* Dziedzicki, 1889

*Phronia persimilis* Hackman, 1970

*Phronia petulans* Dziedzicki, 1889

*Phronia portschinskyi* Dziedzicki, 1889

*Phronia siebeckii* Dziedzicki, 1889

*Phronia signata* Winnertz, 1864

= *austriaca* Winnertz, 1864

*Phronia spinigera* Hackman, 1970

*Phronia strenua* Winnertz, 1864

= *flavicollis* Winnertz, 1864 nom. dubium

*Phronia subsilvatica* Hackman, 1970

*Phronia sudetica* Dziedzicki, 1889

*Phronia sylvatica* Dziedzicki, 1889

*Phronia taczanowskyi* Dziedzicki, 1889

*Phronia tenuis* Winnertz, 1864

*Phronia tiefii* Dziedzicki, 1889

= *marginata* Dziedzicki, 1889

*Phronia triangularis* Winnertz, 1864

*Phronia unica* Dziedzicki, 1889

*Phronia vitrea* Plassmann, 1999

= *carli* Chandler, 2001

*Phronia willistoni* Dziedzicki, 1889

***PLATUROCYPTA*** Enderlein, 1910

*Platurocypta punctum* (Stannius, 1831)

*Platurocypta testata* (Edwards, 1925)

***SCEPTONIA*** Winnertz, 1864

*Sceptonia concolor* Winnertz, 1864

*Sceptonia costata* (van der Wulp, 1859)

*Sceptonia demeijerei* Bechev, 1997

*Sceptonia flavipuncta* Edwards, 1925

*Sceptonia fumipes* Edwards, 1925

*Sceptonia fuscipalpis* Edwards, 1925

*Sceptonia hamata* Ševčík, 2004

*Sceptonia longisetosa* Ševčík, 2004

*Sceptonia membranacea* Edwards, 1925

*Sceptonia nigra* (Meigen, 1804)

*Sceptonia pughi* Chandler, 1991

*Sceptonia regni* Chandler, 1991

*Sceptonia tenuis* Edwards, 1925

*Sceptonia thaya* Ševčík, 2004

***TRICHONTA*** Winnertz, 1864

*Trichonta apicalis* Strobl, 1898

*Trichonta atricauda* (Zetterstedt, 1852)

= *adunca* Edwards, 1925

*Trichonta beata* Gagné, 1981

*Trichonta bicolor* Landrock, 1912

*Trichonta bifida* Lundström, 1909

*Trichonta brevicauda* Lundström, 1909

*Trichonta canora* Gagné, 1981

*Trichonta clara* Gagné, 1981

*Trichonta comica* Gagné, 1981

*Trichonta comis* Gagné, 1981

*Trichonta concinna* Gagné, 1981

*Trichonta conjungens* Lundström, 1909

*Trichonta delicata* Gagné, 1981

*Trichonta eximia* Gagné, 1981

*Trichonta facilis* Gagné, 1981

*Trichonta falcata* Lundström, 1911

*Trichonta fissicauda* (Zetterstedt, 1852)

= *claripennis* Lundström, 1914

*Trichonta flavicauda* Lundström, 1914

*Trichonta fragilis* Gagné, 1981

*Trichonta fusca* Landrock, 1918

*Trichonta generosa* Gagné, 1981

*Trichonta girschneri* Landrock, 1912

*Trichonta hamata* Mik, 1880

*Trichonta icenica* Edwards, 1925

*Trichonta melanura* (Staeger, 1840)

*Trichonta nigritula* Edwards, 1925

*Trichonta palustris* Maximova, 2002

*Trichonta patens* Johannsen, 1912

*Trichonta perspicua* van der Wulp, 1881

= *mediastinalis* (Lundström, 1909)

*Trichonta subfusca* Lundström, 1909

*Trichonta submaculata* (Staeger, 1840)

*Trichonta subterminalis* Zaitzev & Menzel, 1996

*Trichonta terminalis* (Walker, 1856)

* *Trichonta tristis* (Strobl, 1898) (see Notes)

*Trichonta trivittata* Lundström, 1916

*Trichonta venosa* (Staeger, 1840)

*Trichonta vitta* (Meigen, 1830)

*Trichonta vulcani* (Dziedzicki, 1889)

= *appropinquata* (Strobl, 1900)

? = *trifida* Lundström, 1909 (see Notes)

*Trichonta vulgaris* Loew, 1869

= *nigricauda* Lundström, 1906

***ZYGOMYIA*** Winnertz, 1864

*Zygomyia angusta* Plassmann, 1977

*Zygomyia humeralis* (Wiedemann, 1817)

*Zygomyia kiddi* Chandler, 1991

*Zygomyia notata* (Stannius, 1831)

*Zygomyia pictipennis* (Staeger, 1840)

*Zygomyia pseudohumeralis* Caspers, 1980

*Zygomyia semifusca* (Meigen, 1818) (see Notes)

*Zygomyia valida* Winnertz, 1864

*Zygomyia vara* (Staeger, 1840)

*Zygomyia zaitzevi* Chandler, 1991

## Excluded species

Bolitophilidae

*Bolitophila spinigera* Edwards, 1925 misidentified

Keroplatidae

*Macrocera nigropicea* Lundström, 1906 not found within present borders

*Macrocera sudetica* Landrock, 1924 only females, unidentifiable (see Notes)

*Orfelia bicolor* (Macquart, 1826) only females, unidentifiable (see Notes)

Mycetophilidae

*Allodia triangularis* (Strobl, 1895) misidentified

*Cordyla styliforceps* (Bukowski, 1934) misidentified (see Notes)

*Exechiopsis unguiculata* (Lundström, 1911) not found within present borders

*Leptomorphus quadrimaculatus* Matsumura, 1916 misidentified (see Notes)

*Monoclona mikii* Kertesz, 1898 misidentified (see Notes)

*Mycetophila gratiosa* Winnertz, 1864 misidentified (see Notes)

*Mycetophila lunata* Meigen, 1804 misidentified (see Notes)

*Mycetophila vittipes* Zetterstedt, 1852 misidentified (see Notes)

*Palaeodocosia alpicola* (Strobl, 1895) misidentified (see Notes)

*Pseudexechia trisignata* (Edwards, 1913) not found within present borders

*Pseudorymosia optiva* (Dziedzicki, 1910) misidentified (see Notes)

*Sciophila hebes* Johannsen, 1910 misidentified (see Notes)

*Synplasta excogitata* (Dziedzicki, 1910) misidentified (see Notes)

*Trichonta fidelis* Gagné, 1981 mistake (see Notes)

## Notes

***Cordyla styliforceps* (Bukowski, 1934)** was added to the list of Finnish insects by Silfverberg (2007) with a reference to Polevoi (2001) which most probably based on misidentified species (A.Polevoi, pers. comm).

***Leptomorphus quadrimaculatus* Matsumura, 1916** was added to the list of Finnish insects by Silfverberg (2001) with a reference to Polevoi (1995). This record has later shown to belong to *Leptomorphus subforcipatus* (Zaitzev and Ševčík 2002).

***Macrocera sudetica* Landrock, 1924** and ***Orfelia bicolor* (Macquart, 1826)** (= *basalis* Winnertz, 1864). Listed in Hackman (1980) by the only female specimen located in the collection of the Finnish Museum of Natural History “Diptera Fennica” that cannot be recognised. No further records from Finland.

***Monoclona mikii* Kertesz, 1898**. Listed in Hackman (1980) by erroneously identified male specimen of *Monoclona braueri* and by the female specimen (both are located in “Diptera Fennica”) that cannot be recognized. Later this species has been recorded from Finland under its valid name, *Monoclona silvatica* (Jakovlev et al. 2014).

***Mycetophila gratiosa* Winnertz, 1864**. Listed in Hackman (1980) by erroneously identified male specimens of *Mycetophila luctuosa* located in “Diptera Fennica”.

***Mycetophila lunata* Meigen, 1804** was interpreted broadly in old sources, and although it was confirmed by Silfverberg (2001) with a reference to Polevoi (1995) this record has later shown to belong to another related species, *Mycetophila sublunata* Zaitzev, 1999. Hence, *Mycetophila lunata* still needs to be confirmed from Finland.

***Mycetophila vittipes* Zetterstedt, 1852**. Listed in Hackman (1980) by erroneously identified male specimens of *Mycetophila vittipes*-group located in “Diptera Fennica”.

***Palaeodocosia alpicola* (Strobl, 1895)** Listed in Hackman (1980) by erroneously identified male specimen of *Palaeodocosia vittata* located in “Diptera Fennica”.

***Pseudorymosia optiva* (Dziedzicki, 1910)**. The small genus *Pseudorymosia* contains only two known Palaearctic species, *Pseudorymosia fovea* (Dziedzicki, 1910) and *Pseudorymosia optiva* (Dziedzicki, 1910), that in fact might be only one species (Zaitzev 2003). Re-examination of specimens in the “Diptera Fennica” collections formerly identified as *Pseudorymosia optiva* and published by Hackman (1980) revealed that all of them are in fact the widely distributed *Pseudorymosia fovea* as interpreted in newer determination sources (Jakovlev et al. 2006).

***Sciophila hebes* Johannsen, 1910**. Recorded by Komonen (2001) by erroneously identified specimens of *Sciophila antiqua*.

***Synplasta excogitata* (Dziedzicki, 1910)**. Listed in Hackman (1980) by erroneously identified male specimen of *Synplasta gracilis*.

***Trichonta fidelis* Gagné, 1981**. Listed in Hackman (1980) with the note that *Trichonta fidelis* Gagné was only a manuscript of Gagné (1981) at the time. Apparently Gagné changed his mind before publication and used this name for an oriental species instead (Hackman 1982).

***Zygomyia semifusca* (Meigen, 1818)**. Listed in Hackman (1980) as *Mycetophila semifusca*.

### Doubtful and unrecognized species

* ***Keroplatus tuvensis* Zaitzev, 1991**. This species might be a junor synonym of *Keroplatus testaceus* (Kjærandsen et al. 2007). With this reference, it was deleted from the list of Finnish insects by Silfverberg (2012), but this synonym has not been adopted in the checklist of North European fungus gnats (Kjærandsen 2012). Hereby we keep *Keroplatus tuvensis* in the Finnish checklist pending revision of the *Keroplatus testaceus* complex of species.

* ***Mycetophila biformis* Maximova, 2002** preocc. Added by Jakovlev et al. (2014). This is a junior primary homonym of *Mycetophila biformis* Duret, 1992. A replacement name will be given by the author in the near future (Yulia Maximova, pers. comm.).

* ***Trichonta tristis* (Strobl, 1898)**. Judging from illustration of Dziedzicki (1889), he actually illustrated this species as *Trichonta vulcani*. This makes *vulcani* a junior synonym of *tristis*. In this case, *Trichonta trifida* Lundström, 1909 becomes the valid species that we now call *vulcani* (J. Kjærandsen, pers.comm.). Here we list *Trichonta vulcani* and *Trichonta tristis* as valid species sensu Zaitzev (2003), figures 107,1 and 109,5.

### Species listed by Hackman (1980) with a question marks

Five species, ***Docosia flavicoxa* Strobl, 1900**, ***Cordyla bergensis* (Barendrecht, 1938)**, ***Cordyla murina* Winnertz, 1864**, ***Mycetophila lunata* Meigen, 1804** and ***Phronia petulans* Dziedzicki, 1889** were listed in the former checklist of Finnish Diptera (Hackman 1980) with a question marks. Later, Finnish records were confirmed for *Docosia flavicoxa* by Jakovlev et al. (2014), for *Cordyla murina* by Jakovlev et al. (2006), and for *Phronia petulans* by Jakovlev and Polevoi (2009). *Cordyla bergensis* was excluded as a junior synonym of *Cordyla nitidula* (Jakovlev et al. 2006). *Mycetophila lunata* – see above.
